# Circulating Trimethylamine N-Oxide Is Associated with Increased Risk of Cardiovascular Mortality in Type-2 Diabetes: Results from a Dutch Diabetes Cohort (ZODIAC-59)

**DOI:** 10.3390/jcm10112269

**Published:** 2021-05-24

**Authors:** Jose L. Flores-Guerrero, Peter R. van Dijk, Margery A. Connelly, Erwin Garcia, Henk J. G. Bilo, Gerjan Navis, Stephan J. L. Bakker, Robin P. F. Dullaart

**Affiliations:** 1 Department of Internal Medicine, Division of Nephrology, University of Groningen, University Medical Center Groningen, 9700 RB Groningen, The Netherlands; g.j.navis@umcg.nl (G.N.); s.j.l.bakker@umcg.nl (S.J.L.B.); 2 Department of Internal Medicine, Division of Endocrinology, University of Groningen, University Medical Center Groningen, 9700 RB Groningen, The Netherlands; p.r.van.dijk@umcg.nl (P.R.v.D.); dull.fam@12move.nl (R.P.F.D.); 3 Diabetes Centre, Isala, 8025 AB Zwolle, The Netherlands; 4 Laboratory Corporation of America Holdings (LabCorp), Morrisville, NC 27560, USA; connem5@labcorp.com (M.A.C.); garce14@labcorp.com (E.G.); 5 Department of Internal Medicine, Division of Internal Medicine, Faculty of Medicine, University of Groningen, 9713 AM Groningen, The Netherlands; h.j.g.bilo@umcg.nl

**Keywords:** biomarker, BCAA, cardiovascular disease, trimethylamine-N-oxide, TMAO, type 2 Diabetes

## Abstract

Trimethylamine N-oxide (TMAO), a novel cardiovascular (CV) disease and mortality risk marker, is a gut microbiota-derived metabolite as well. Recently, plasma concentrations of branched-chain amino acids (BCAA) have been reported to be affected by microbiota. The association of plasma TMAO with CV mortality in Type 2 Diabetes (T2D) and its determinants are still incompletely described. We evaluated the association between plasma BCAA and TMAO, and the association of TMAO with CV mortality in T2D individuals. We used data of 595 participants (mean age 69.5 years) from the Zwolle Outpatient Diabetes project Integrating Available Care (ZODIAC) cohort were analyzed. Plasma TMAO and BCAA were measured with nuclear magnetic resonance spectroscopy. CV mortality risk was estimated using multivariable-adjusted Cox regression models. Cross-sectionally, TMAO was independently associated with BCAA standardized (Std) β = 0.18 (95% Confidence Interval (CI) 0.09; 0.27), *p* <0.001. During a median follow-up of 10 years, 113 CV deaths were recorded. In Cox regression analyses, adjusted for multiple clinical and laboratory variables including BCAA, TMAO was independently associated with CV mortality: adjusted hazard ratio (_adj_HR) 1.93 (95% CI 1.11; 3.34), *p* = 0.02 (for the highest vs. the lowest tertile of the TMAO distribution). The same was true for analyses with TMAO as continuous variable: _adj_HR 1.32 (95% CI 1.07; 1.63), *p* = 0.01 (per 1 SD increase). In contrast, BCAAs were not associated with increased CV mortality. In conclusion, higher plasma TMAO but not BCAA concentrations are associated with an increased risk of CV mortality in individuals with T2D, independent of clinical and biochemical risk markers.

## 1. Introduction

Trimethylamine-N-oxide (TMAO) is a microbiota-derived metabolite [1,2] that recently has gained attention as a consequence of its potential role in the progress of ischemic heart disease [3,4] kidney disease [5,6,7], complications in the setting of Type 2 Diabetes (T2D) [8,9] and premature mortality in the general population [10]. Trimethylamine (TMA) is a by-product of a microbial fermentation, in which the gut microbiota metabolizes dietary components such as phosphatidylcholine, choline, and L-carnitine to be used as carbon fuel supply by the gut microbiota. Subsequently, the conversion of TMA to TMAO occurs in the host liver by the flavin monooxygenase 3, after which it is cleared by the kidneys [11].

Even though some studies have identified an association of plasma concentrations of TMAO with adverse cardiovascular (CV) outcomes [10], studies in individuals with latent comorbidities are not always consistent [5]. Moreover, the association of TMAO with CV mortality in individuals with T2D has recently been identified to be present in high-risk individuals [12].

Recently, it has been shown that plasma concentrations of branched-chain amino acids (BCAA) are also affected by the microbiota in both animal models and humans [13]. Moreover, it has been hypothesized that the contribution of the microbiota on BCAA concentrations may be more important on settings related to insulin resistance, such as T2D [14]. Therefore, the aim of the present study was to evaluate the association of circulating BCAA concentrations with concentrations of TMAO and the determine the potential association of TMAO with CV taking account concentrations of BCAA in a prospective cohort of outpatients with T2D.

## 2. Materials and Methods

### 2.1. Study Population and Data Collection

Briefly, the Zwolle Outpatient Diabetes project Integrating Available Care (ZODIAC) study is a Dutch cohort of patients with T2D from the Zwolle region in the northern part of the Netherlands. The design of the ZODIAC study has been described in detail elsewhere [15]. Briefly, the ZODIAC study was initiated in 1998, in this study, the effects of a shared-care project in a primary care population of patients with T2D were investigated. At the beginning of the prospective cohort study 1143 patients with T2D were enrolled; patients with a reduced life expectancy, i.e., with insufficient cognitive abilities or active cancer were excluded from participation. For the current study, subjects with missing data on outcome, and those with missing quantification of TMAO or BCAA by means of nuclear magnetic resonance (NMR) at baseline were excluded, leaving 594 subjects for the present analyses. This report follows the Strengthening the Reporting of Observational Studies in Epidemiology (STROBE) reporting guideline (Supplementary Table S1). The ZODIAC study and the informed consent procedure was approved by the local medical ethics committee of the Isala Clinics, Zwolle, the Netherlands (Medisch Ethische Toetsingscommissie (METC) reference numbers 03.0316 and 07.0335). Informed consent was obtained for all patients by the participating diabetes specialist nurses and the consent was documented in the patient records. All procedures were conducted according to the Declaration of Helsinki [16]. 

During visits to the outpatient clinic, all baseline data were collected as previously described [15]. Blood pressure was measured twice with a Welch Allyn sphygmomanometer (Skaneateles Falls, NY, USA) in the supine position after at least 5 min of rest. Pulse pressure was determined as the difference between systolic and diastolic blood pressure. Height and weight were measured with the participants standing without shoes and heavy outer garments. Body mass index (BMI) was calculated by dividing weight in kilograms by height in meters squared.

Baseline data included a medical history of cardiovascular disease (CVD), tobacco consumption, and use of medication, were collected during the annual check-up of the patient by the general practitioner or practice nurse. Patients were considered to have a history of macrovascular complications if they had a history of stroke, angina pectoris, myocardial infarction, transient ischemic attack, percutaneous transluminal coronary angioplasty, coronary artery bypass grafting, or peripheral vascular disease.

Microvascular complications were defined as the presence of one or more of the following entities: Neuropathy was defined as two or more errors out of three tests of foot sensibility, using a 5.07 Semmes-Weinstein monofilament, at least at one foot: diabetic retinopathy was investigated with a retinal camera, and the fundus photos were judged by an ophthalmologist: nephropathy was defined as eGFR <60 mL/min/1.73m^2^ and/or albuminuria, which was defined as an albumin-to-creatinine ratio >3.5 mg/mmol for women and >2.5 mg/mmol for men. 

### 2.2. Clinical Endpoint

The primary end point was CV mortality. In 2013, vital status and cause of death were retrieved from records maintained by the hospital and the general practitioners or from the Municipal Personal Records Database. Causes of death were coded according to the International Classification of Diseases, Ninth Revision. Cardiovascular death was defined as death in which the principal cause of death was cardiovascular in nature, using International Classification of Diseases, Ninth Revision codes 390 to 459 [17,18].

### 2.3. Laboratory Measurements

Laboratory assessment included non-fasting lipid profile, glycated hemoglobin (HbA1c), serum creatinine, urinary albumin-to-creatinine ratio (ACR), and blood pressure. Serum creatinine was measured by a kinetic colorimetric Jaffe method (Modular P Analyzer; Roche, Almere, the Netherlands), The creatinine-based Chronic Kidney Disease Epidemiology Collaboration (CKD-EPI) equation was used to estimate glomerular filtration rate (eGFR) [19]. To calculate the eGFR, serum creatinine levels were reduced by 5%, because serum creatinine measurements in this study were not standardized to isotope dilution mass spectrometry [20]. Urinary albumin was measured using immunonephelometry (Behring Nephelometer, Mannheim, Germany).

TMAO and BCAA were measured in EDTA-anticoagulated plasma samples using a Vantera^®^ Clinical Analyzer (LabCorp, Morrisville, NC, USA), a fully automated, high-throughput, 400 MHz proton (1H) nuclear magnetic resonance (NMR) spectroscopy platform. TMAO were quantified from one-dimensional (1D) proton (1H) Carr–Purcell–Meiboom–Gill (CPMG) spectra by means of deconvolution assays as previously described [21,22]. The TMAO assay has intra- and inter-assay coefficients of variation (CV%) range from 4.3–10.3% and 9.8–14.5%, respectively, and a limit of quantitation of 3.3 μM. 

The validation of the use of NMR for quantification of BCAA has been previously described by our group [23,24]. Briefly, coefficients of variation for inter- and intraassay precision ranged from 1.8% to 6.0%, 1.7% to 5.4%, 4.4% to 9.1%, and 8.8% to 21.3%, for total BCAA, valine, leucine, and isoleucine, respectively. BCAA quantified from the same samples using NMR and LC-MS/MS were highly correlated, showing a *r*^2^ = 0.97, 0.95 and 0.90 for valine, leucine, and isoleucine, respectively [23,24].

### 2.4. Statistical Analysis

Normally distributed data were presented as mean and standard deviation, whereas skewed data were expressed as median and interquartile range. Categorical data were presented as number and percentage. Skewed data were log-transformed when appropriate. Linear trends across TMAO tertiles were determined using ANOVA for normally distributed data, Kruskal-Wallis test for skewed distributed data, and χ^2^ test for categorical variables. For the cross-sectional analysis, a multivariable linear regression analysis was performed using the plasma concentration of TMAO as dependent variable. Given the fact that eGFR is calculated using creatinine and age information, eGFR was not included in the cross-sectional analysis. Therefore, we were able to better evaluate the association of age and creatinine with plasma concentrations of TMAO. To further investigate the potential association of TMAO with BCAA, as suggested by previous research [13,14], we included total BCAA.

For the prospective analysis, we plotted cumulative Kaplan-Meier curves for risk of CV mortality during follow-up according to tertiles of TMAO. Time-to-event Cox proportional hazards models were used to compute hazard ratios (HRs) and 95% CI of CV mortality risk among the 594 participants. HRs were calculated in models adjusted for age, sex, T2D duration, smoking behavior, prevalent macrovascular complications, systolic blood pressure, HbA1c, total cholesterol, high-density lipoprotein cholesterol (HDL-cholesterol), triglycerides, albuminuria and reduced eGFR at baseline (<60 mL/min/1.73 m^2^). The Cox proportional hazard assumption was tested through the evaluation of independence between scaled Schoenfeld residuals with time for each variable and for every model as a whole; this assumption was met, with no indication for a violation [25]. To further evaluate the robustness of the association and the risk of bias, a sensitivity analysis was conducted to calculate the Robustness of Inference to Replacement [26].

The net reclassification improvement (NRI) [27] was calculated to evaluate whether the inclusion of TMAO into a model can improve the risk reclassification of participants. As a base model, a conventional model for cardiovascular mortality [28] already used to evaluate the NRI for mortality in people with T2D was used. Such model includes age, sex, smoking behavior, systolic blood pressure, total cholesterol, HDL-cholesterol, and antihypertensive medications. For the computation of NRI three predefined risk categories of cardiovascular mortality previously described in the literature were used, i.e., low (<7%), medium (7% to 20%), and high (>20%) [28]. All statistical analyses were performed with R language for statistical computing software [29], v. 4.0.2.

## 3. Results

### 3.1. Baseline Characteristics

Out of the 594 subjects with available measurements of TMAO that were included in the current study, the mean age of the population was 69.5 ± 11.2 years and 42.1% (*n* = 250) were men. The median (IQR) plasma total TMAO concentration was 3.9 (2.4–6.5) μmol/L. Participant characteristics at baseline are shown in Table 1. Subjects with higher TMAO concentrations were more likely to be older, have higher systolic blood pressure and lower diastolic blood pressure, have a pronounced increased prevalence of macrovascular complications, have a slightly increased prevalence of microvascular complications and albuminuria as well as a lower eGFR (Table 1).

### 3.2. Cross-Sectional Analyses

The association of the concentration of TMAO with baseline characteristics was evaluated with multivariable linear regression analysis (Table 2). In a multivariable model, including the variables presented in Table 1, TMAO remained positively associated with systolic blood pressure (Std. β = 0.27 (95% CI 0.17; 0.38), *p* < 0.001), and negatively associated with diastolic blood pressure (Std. β = −0.19 (95% CI −0.29; −0.09), *p* < 0.001) (Supplementary Figure S1). TMAO was also associated with microvascular complications (Std. β = 0.23 (95% CI 0.06; 0.41), *p* = 0.009), total cholesterol, (Std. β = 0.12 (95% CI 0.02; 0.22), *p* = 0.03), BCAAs (Std. β = 0.18 (95% CI 0.09; 0.27), *p* < 0.001) and serum creatinine (Std. β = 0.17 (95% CI 0.09; 0.26), *p* < 0.001). Those results were comparable to the coefficients calculated in univariable regression analyses. (Supplementary Table S2). Moreover, higher plasma concentrations of TMAO were positively associated with greater pulse pressure (Std. β = 0.10 (95% CI 0.06; 0.14), *p* <0.001) (Supplementary Figure S2).

### 3.3. Longitudinal Analyses on TMAO and CV Mortality

**Figure 1:**
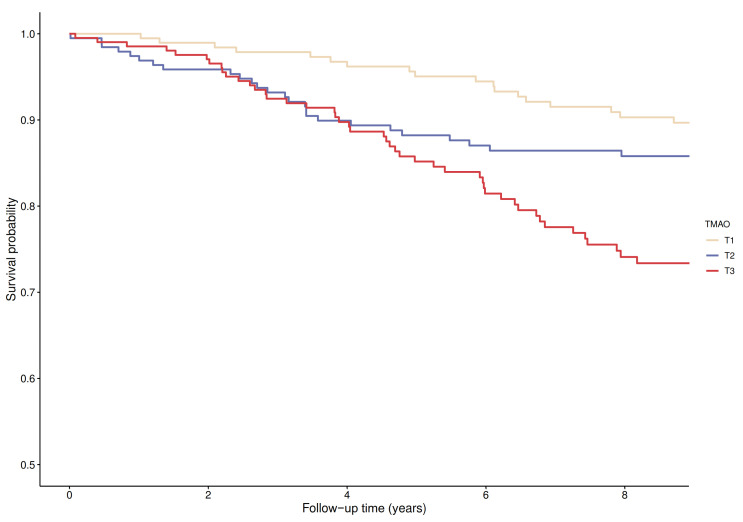
Kaplan-Meier plot for cardiovascular mortality comparing tertiles of TMAO (log-rank test, *p* < 0.001).

After a median (IQR) follow-up of 10.4 (5.7–11.8) years, 113 deaths attributed to cardiovascular disease were recorded. Kaplan-Meier curves for cardiovascular mortality according to tertiles of TMAO plasma concentration are presented in Figure 1. There was an increased risk of cardiovascular mortality associated with the top tertile of TMAO concentrations (*p* for log-rank test <0.001).

**Figure 2:**
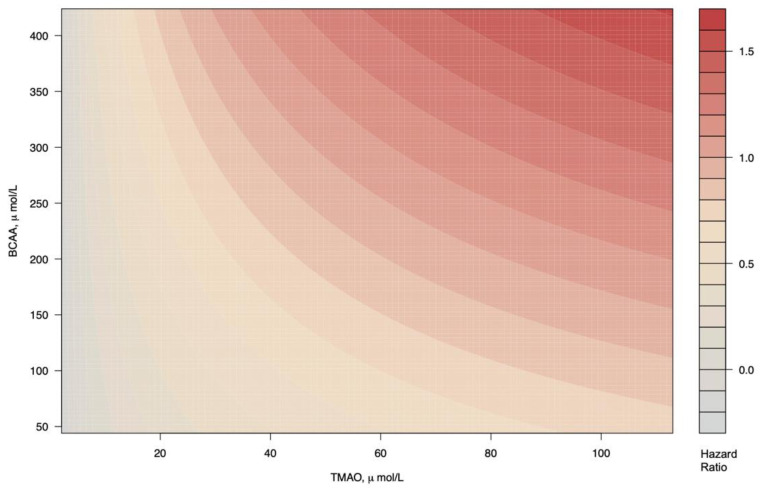
Cardiovascular mortality as a function of TMAO and BCAA in T2D. Subjects with highest concentrations of both BCAA and TMAO present higher risk of cardiovascular mortality.

In Cox proportional hazard regression analyses that examined the TMAO as HR per 1 Ln SD, increased plasma concentrations of TMAO were associated with increased risk of cardiovascular mortality independent of age and sex (adjusted HR, 1.39 (95% CI 1.16; 1.67), *p* < 0.001, model 1, Table 3); T2D duration, smoking and prevalent macrovascular complications (_adj_HR, 1.29 (95% CI 1.07; 1.56), *p* = 0.007, model 2, Table 3); systolic blood pressure, HbA1c, total cholesterol, HDL-cholesterol, triglycerides and total BCAA (_adj_HR, 1.26 (95% CI 1.04; 1.54), *p* = 0.02, model 3, Table 3); albuminuria (_adj_HR, 1.27 (95% CI 1.03; 1.56), *p* = 0.02, model 4, Table 3) and reduced eGFR (<60 mL/min/1.73 m^2^) (_adj_HR, 1.32 (95% CI 1.07; 1,63) *p* = 0.01, model 5, Table 3). There was a marginally significant interaction between TMAO and BCAA (*p* = 0.05), which is graphically depicted in Figure 2. The proportional hazards assumptions were not violated for any of the variables in the Cox regression models. The analyses of plasma concentration of TMAO as a categorical variable, using the first tertile as the reference group, showed that the third tertile of TMAO plasma concentration was also associated with higher risk of cardiovascular mortality in all the Cox regression models described, resulting in a fully _adj_HR 1.93 (95% CI 1.11; 3.34), *p* = 0.02 (Table 3). According to the sensitivity analyses, to invalidate the inference about the association of TMAO with CVD mortality, 57.4% of the estimated effect would have to be due to bias. Likewise, to invalidate the inference, in 341 out of the 594 participants the effect of TMAO on cardiovascular mortality should be 0.

The addition of TMAO to a model for cardiovascular mortality [28], which included age, sex, smoking behavior, systolic blood pressure, total cholesterol, HDL-cholesterol, and antihypertensive medications, allowed the reclassification of 21% of the sample. Seven percent of the participants in the low-risk category were correctly reclassified to medium-risk and 14% of the participants in the middle risk category were correctly reclassified to the high-risk category. The improvement in the classification of participants into predicted risk categories was statistically significant with a NRI of 0.24 (95% CI 0.03; 0.44; *p* = 0.02).

### 3.4. Longitudinal Analysis on BCAA and CV Mortality

Plasma concentrations of BCAAs were not independently associated with increased risk of mortality, neither when analyzed per 1 Ln SD (_adj_HR 0.90 (95% CI 0.72; 1.12), *p* = 0.19), nor when analyzed as tertiles, using the first tertile as the reference group (_adj_HR 0.88 (95% CI 0.52; 1.49), *p* = 0.64) (Supplementary Table S3). 

## 4. Discussion

In this prospective study, we have shown that higher circulating TMAO concentrations were associated with an increased risk of cardiovascular mortality in individuals with T2D. TMAO remained significantly associated, after the adjustment for several CVD risk markers, history of macrovascular complications, and circulating concentrations of BCAAs. Moreover, addition of circulating concentrations of TMAO to a base model of cardiovascular mortality risk, improved the patient reclassification from a lower to a higher risk category. In cross-sectional analyses, plasma concentrations of TMAO at baseline were associated with renal function, blood pressure, and plasma BCAA.

As expected [7], plasma TMAO was associated with creatinine, reflecting accumulation of TMAO in the context of impaired renal function [10]. Moreover, systolic and diastolic blood pressure respectively displayed strong positive and negative associations with plasma concentrations of TMAO, resembling the increase of systolic and the decrease of diastolic blood over adulthood [30]. The association of systolic and diastolic blood pressure with plasma concentrations of TMAO were independent of age, and the decline of the diastolic blood pressure is more evident in the group with high concentration of TMAO (Supplementary Figure S1). Further analysis demonstrated that higher plasma concentrations of TMAO were associated with elevated pulse pressure (Supplementary Figure S2), probably reflecting an association of TMAO with arterial stiffness. Although this association cannot provide a causal link, it has been recently reported that TMAO supplementation in rats induced an aging-like artery dysfunction, via dysregulation of endothelium-dependent dilation [31]. Different mechanisms are involved in the deleterious effect of TMAO: oxidative stress characterized by excess of nitrotyrosine, endothelial nitric oxide synthase, and impaired nitric oxide-mediated dilation. Importantly, such mechanisms were confirmed in human endothelial cells [31]. Those findings were in line with the results from this cohort study. Of further note, experimental studies have provided evidence about the importance and causal role of TMAO in cardiovascular disease [11]. It has been proposed that the role of TMAO in the risk of cardiovascular disease could be mediated by several pathways, such as the acceleration of atherosclerosis by enhancing the formation of foam cells and atherosclerotic plaques [1], the inhibition of reverse cholesterol process whereby cholesterol is transported form the arterial wall back to the liver where it is metabolized and excreted in the bile [2], as well as the platelet hyperactivity, nevertheless the entire mechanisms are not fully understood [32]. Such biological background provides a rationality to the growing body of epidemiological evidence about the association of TMAO with cardiovascular mortality, summarized in a meta-analysis that included 19, 256 subjects. In such meta-analysis subjects with high concentrations of TMAO had a higher relative risks of with major adverse cardiovascular events [33].

The role of gut microbiota-derived metabolites in the development of cardiovascular disease has gained recent attention [11], particularly in individuals with T2D [34]. Nevertheless, the association of TMAO with risk of cardiovascular mortality in subjects with T2D has not being sufficiently studied. To the best of our knowledge, there is only one study that reported on such an association in a time-to-event analysis. Croyal and colleagues reported that T2D patients with high concentrations of TMAO presented a higher risk of mortality during a seven year follow-up [8]. The reported association remained after adjustment for several confounding factors. However, the concentrations of TMAO were only analyzed as a categorical variable [8], while in our study, TMAO was analyzed both as a categorical and as a continuous variable, which improves the robustness of the analysis [35].

### Strengths and Limitations 

This study has some strengths. First, this study comprises a long-term follow-up, and includes the record of several important confounders. The study population consisted of T2D patients exclusively treated in primary care setting, and therefore those results could be extrapolated to patients in real practice. Several limitations of the present study deserve mention. First, the present study was conducted in the north of the Netherlands, and mainly comprises individuals of Caucasian ancestry, which could limit the extrapolation of our findings to other ethnicities. Likewise, there was no available data on dietary patters, which is major contributor of TMAO plasma concentration, therefore it was not possible to further evaluate if the association was independent of any particular dietary pattern, particularly those which are closely related to cardiovascular disease and high concentrations of TMAO production, such as meat-rich diets [2]. In addition, patients whose markers were not measured, where excluded from the analyses, which could lead to selection bias; nevertheless, those missing values were missing completely at random. Importantly, various other uremic toxins have been described to be involved in the development of cardiovascular disease, i.e., p-cresyl sulfate and indoxyl sulfate [36], it remains to be explored how important is role of TMAO in comparison with other gut-derived uremic toxins in the prediction of CV related outcomes in T2D subjects. Finally, the association of TMAO with BCAAs, was apparently non-linear (Table 1); probably due to the fact that circulating concentrations of TMAO does not reflects the whole array of microbiota metabolism. A more comprehensive study with a wider range of microbiota-derived biomarkers is desirable to better depict the association of BCAAs with the microbiota. Likewise, considering that the BCAA are closely related with T2D and insulin resistance [23], which is a major risk factor for CVD; therefore, it may be possible the association between BCAA and risk of cardiovascular mortality may be neglected in an analysis restricted to patients with a history of T2D.

In conclusion, this prospective T2D cohort study indicates that that high concentrations of circulating TMAO were associated with a higher risk of cardiovascular mortality, independent of traditional risk factors. In contrast, BCAA, were not associated with cardiovascular mortality, although the highest mortality was found in patients with high TMAO and high BCAA. Further investigation is needed to determine whether TMAO is a potential treatment target in individuals with T2D to possibly reduce the risk of cardiovascular mortality.

## Figures and Tables

**Table 1:** Baseline participant characteristics according to tertiles of plasma TMAO concentration.

Characteristic	All (*n* = 594)	Tertile 1 (*n* = 194)	Tertile 2 (*n* = 193)	Tertile 3 (*n* = 207)	*p* Value *
Men, *n* (%)	250 (42.1%)	94 (48.5%)	74 (38.3%)	82 (39.6%)	0.08
Age, years	69.45 (11.19)	66.93 (11.30)	68.64 (11.60)	72.55 (9.93)	<0.001
BMI, kg/m^2^	28.84 (4.53)	28.73 (4.29)	29.12 (4.51)	28.69 (4.77)	0.58
SBP, mmHg	154.40 (24.02)	151.06 (24.68)	156.39 (22.48)	155.68 (24.57)	0.05
DBP, mmHg	83.38 (10.88)	83.55 (11.24)	85.17 (10.32)	81.54 (10.82)	0.004
Never smoked, *n* (%)	123 (20.7%)	37 (19.1%)	47 (24.4%)	39 (18.8%)	0.31
Diabetes duration, years	4.62 (2.00, 9.11)	4.00 (2.0, 8.0)	5.00 (2.0, 10.0)	5.00 (2.0, 11.0)	0.58
Macrovascular comp., *n* (%)	220 (37.0%)	69 (35.6%)	60 (31.1%)	91 (44.0%)	0.02
Microvascular comp., *n* (%)	136 (48.4%)	44 (45.8%)	44 (44.9%)	48 (55.2%)	0.31
Glucose, mg/dL	155.62 (63.15)	157.99 (60.02)	157.96 (64.88)	151.22 (64.44)	0.24
HbA1c, %	7.32 (1.26)	7.28 (1.35)	7.35 (1.26)	7.33 (1.19)	0.88
TMAO, μmol/L	3.90 (2.40, 6.50)	1.90 (1.30, 2.40)	3.90 (3.30, 4.50)	8.20 (6.30, 11.75)	<0.001
TC, mmol/L	5.58 (1.12)	5.53 (1.13)	5.59 (0.98)	5.61 (1.23)	0.76
HDL-C, mmol/L	1.11 (0.93, 1.36)	1.10 (0.92, 1.37)	1.11 (0.93, 1.37)	1.14 (0.95, 1.35)	0.69
Triglycerides, mmol/L	2.55 (1.50)	2.58 (1.58)	2.64 (1.59)	2.44 (1.34)	0.38
BCAA, μmol/L	512.94 (126.69)	510.97 (120.61)	508.90 (125.24)	518.55 (133.80)	0.72
Valine, μmol/L	273.47 (54.40)	274.56 (52.37)	273.26 (53.50)	272.65 (57.28)	0.93
Leucine, μmol/L	171.21 (53.46)	169.80 (49.66)	166.44 (51.17)	176.98 (58.47)	0.13
Isoleucine, μmol/L	68.27 (30.03)	66.63 (28.17)	69.22 (30.34)	68.93 (31.48)	0.64
Serum creatinine, mmol/L	94.16 (18.94)	90.09 (15.09)	92.18 (16.33)	99.81 (22.79)	<0.001
eGFR, mL/1.73 m^2^/min) ^2^	68.44 (18.80)	72.37 (17.66)	71.22 (18.29)	62.16 (18.76)	<0.001
Albuminuria, *n* (%)	194 (33.9%)	49 (25.9%)	70 (37.6%)	75 (37.9%)	0.02

Abbreviations: BCAA, Branched-chain amino acids; BMI, body mass index; DBP, diastolic blood pressure; eGFR, estimated glomerular filtration rate; HbA1c glycated hemoglobin; HDL-C, high-density lipoprotein cholesterol; SBP, systolic blood pressure; TC, total cholesterol; TMAO, Trimethylamine N-Oxide. Values are shown as mean (standard deviation) or median (interquartile range), accordingly. *p* values were determined using ANOVA for normally distributed data, Kruskal-Wallis test for skewed distributed data, and χ2 test for categorical variables. * Correspond to the comparison between all groups.

**Table 2:** Multivariable associations of baseline characteristics with plasma concentrations of TMAO.

Variable	Std β (95% CI)	*p* Value
Men, (yes)	−0.17 (−0.36; 0.01)	0.06
Age, years	−0.04 (−0.13; 0.06)	0.45
BMI, kg/m^2^	0.00 (−0.08; 0.08)	0.98
SBP, mmHg	0.27 (0.17; 0.38)	**<0.001**
DBP, mmHg	−0.19 (−0.29; −0.09)	**<0.001**
Never smoked, (yes)	−0.07 (−0.28; 0.13)	0.47
Diabetes duration, years	−0.05 (−0.13; 0.03)	0.24
Macrovascular comp., (yes)	0.11 (−0.06; 0.28)	0.22
Microvascular comp., (yes)	0.23 (0.06; 0.41)	**0.009**
Glucose, mg/dL	−0.04 (−0.13; 0.05)	0.35
HbA1c, %	−0.01 (−0.11; 0.08)	0.75
TC, mmol/L	0.12 (0.02; 0.22)	**0.02**
HDL-C, mmol/L	−0.02 (−0.11; 0.07)	0.69
Triglycerides, mmol/L	−0.10 (−0.19; 0.00)	0.05
BCAA, μmol/L	0.18 (0.09; 0.27)	**<0.001**
Serum creatinine, mmol/L	0.17 (0.09; 0.26)	**<0.001**
Albuminuria, (yes)	−0.10 (−0.28; 0.08)	0.28

Standardized regression coefficients are shown. Abbreviations: BCAA, Branched-chain amino acids; BMI, body mass index; DBP, diastolic blood pressure; HbA1c, glycated hemoglobin; HDL-C, high-density lipoprotein cholesterol; SBP, systolic blood pressure; TC, total cholesterol. *p* values <0.05 are highlighted with bold font.

**Table 3:** Association of TMAO with cardiovascular mortality, assessed with Cox Proportional Hazard ratios.

	TMAO per 1 Ln SD Increment		T1	T2		T3	
Participants, *n*	595		194	193		208	
Events, *n*	113		22	32		59	
	HR (95% CI)	*p* value		HR (95% CI)	*p* value *	HR (95% CI)	*p* value **
Crude Model	1.58 (1.33; 1.87)	<0.001	(ref)	1.61 (0.94; 2.77)	0.08	3.26 (1.99; 5.34)	<0.001
Model 1	1.39 (1.16; 1.67)	<0.001	(ref)	1.49 (0.86; 2.56)	0.15	2.18 (1.33; 3.58)	0.002
Model 2	1.29 (1.07; 1.56)	0.007	(ref)	1.58 (0.91; 2.72)	0.10	2.06 (1.25; 3.41)	0.004
Model 3	1.26 (1.04; 1.54)	0.02	(ref)	1.42 (0.82; 2.46)	0.21	1.87 (1.12; 3.12)	0.02
Model 4	1.27 (1.03; 1.56)	0.02	(ref)	1.39 (0.78; 2.48)	0.26	1.92 (1.12; 3.30)	0.02
Model 5	1.32 (1.07; 1.63)	0.01	(ref)	1.30 (0.73; 2.32)	0.38	1.93 (1.12; 3.35)	0.02

Data are presented as hazard ratios (HRs) with 95% confidence intervals (CIs) and *p* values. *p* values correspond to the comparison between T1 vs T2 (*) and T1 vs T3 (**). Model 1. Model adjusted for age +sex. Model 2. Model 1 + T2D duration + smoking + macrovascular complications. Model 3. Model 2 + SBP + HbA1c + TC + HDL-C+ TG. Model 4. Model 3 + albuminuria. Model 5. Model 4 + reduced eGFR (<60 mL/min/1.73 m^2^) + total BCAA. Abbreviations: BCAA, Branched-chain amino acids; eGFR, estimated glomerular filtration rate; HbA1c, glycated hemoglobin; HDL-C, high-density lipoprotein cholesterol; SBP, systolic blood pressure; T2D, Type 2 Diabetes; TC, total cholesterol; TG, triglycerides. (ref) indicates the group of reference.

## Data Availability

The dataset used during the current study are available from the corresponding author on reasonable request.

## Supplementary Materials

The following are available online at https://www.mdpi.com/article/10.3390/jcm10112269/s1, Supplementary Table S1. STROBE Statement—Checklist of items that should be included in reports of cohort studies. Supplementary Table S2. Univariable associations of baseline characteristics with plasma concentrations of TMAO. Supplementary Table S3. Association of branched-chain amino acids with cardiovascular mortality, assessed with Cox Proportional Hazard ratios. Supplementary Figure S1. Association of age with blood pressure by plasma concentrations of Trimethylamine N-oxide. Supplementary Figure S2. Association of pulse pressure with plasma concentrations of Trimethylamine N-oxide.
