# Supplementary material for: Circulating Trimethylamine N-Oxide Is Associated with Increased Risk of Cardiovascular Mortality in Type-2 Diabetes: Results from a Dutch Diabetes Cohort (ZODIAC-59)
Source: J Clin Med. 2021 May 24;10(11):2269. doi: 10.3390/jcm10112269 (PMC8197378; doi:10.3390/jcm10112269)
Supplement: Supplementary file 1 [file jcm-10-02269-s001.zip › jcm-1217209-supplementary.pdf]

Journal of
Clinical Medicine

MDPI

*Supplementary Materials*

# Circulating Trimethylamine N-Oxide is associated with increased risk of cardiovascular mortality in type 2 diabetes: Results from a Dutch Diabetes Cohort (ZODIAC-59).

**Jose L. Flores-Guerrero[1*], Peter R. van Dijk[2,3], Margery A. Connelly[4], Erwin Garcia[4], Henk J.G. Bilo[5], Gerjan Navis[1], Stephan J.L. Bakker[1] and Robin P.F. Dullaart[2]**

[1] Department of Internal Medicine, Division of Nephrology, University of Groningen, University Medical Center Groningen, 9700 RB, Groningen, The Netherlands; g.j.navis@umcg.nl (G.N.); s.j.l.bakker@umcg.nl (S.J.L.B.)
[2] Department of Internal Medicine, Division of Endocrinology, University of Groningen, University Medical Center Groningen, 9700 RB, Groningen, The Netherlands; p.r.van.dijk@umcg.nl (P.R.v.D.); dull.fam@12move.nl (R.P.F.D.)
[3] Diabetes Centre, Isala, 8025 AB, Zwolle, The Netherlands
[4] Laboratory Corporation of America Holdings (LabCorp), Morrisville, NC 27560, North Carolina, USA; connem5@labcorp.com (M.A.C.); garce14@labcorp.com (E.G.)
[5] Department of Internal Medicine, Division of Internal Medicine, Faculty of Medicine, University of Groningen, 9713 AM, Groningen, The Netherlands; h.j.g.bilo@umcg.nl;
* Correspondence: j.l.flores.guerrero@umcg.nl; Tel: +31-50-3610137

**Supplementary Table S1.** STROBE Statement—Checklist of items that should be included in reports of cohort studies.

| | Item No | Recommendation | Page No |
|---|---|---|---|
| Title and abstract | 1 | (a) Indicate the study's design with a commonly used term in the title or the abstract | 2 |
| | | (b) Provide in the abstract an informative and balanced summary of what was done and what was found | 2 |
| | | Introduction | |
| Background/rationale | 2 | Explain the scientific background and rationale for the investigation being reported | 4 |
| Objectives | 3 | State specific objectives, including any prespecified hypotheses | 4 |
| | | Methods | |
| Study design | 4 | Present key elements of study design early in the paper | 5 |
| Setting | 5 | Describe the setting, locations, and relevant dates, including periods of recruitment, exposure, follow-up, and data collection | 5 |
| Participants | 6 | (a) Give the eligibility criteria, and the sources and methods of selection of participants. Describe methods of follow-up | 5 |
| | | (b) For matched studies, give matching criteria and number of exposed and unexposed | |
| Variables | 7 | Clearly define all outcomes, exposures, predictors, potential confounders, and effect modifiers. Give diagnostic criteria, if applicable | 7,8 |
| Data sources/ measurement | 8* | For each variable of interest, give sources of data and details of methods of assessment (measurement). Describe comparability of assessment methods if there is more than one group | 6,7 |
| Bias | 9 | Describe any efforts to address potential sources of bias | 7,8 |
| Study size | 10 | Explain how the study size was arrived at | 5 |
| Quantitative variables | 11 | Explain how quantitative variables were handled in the analyses. If applicable, describe which groupings were chosen and why | 5, 7 |
| Statistical methods | 12 | (a) Describe all statistical methods, including those used to control for confounding | a.7-8 |
| | | (b) Describe any methods used to examine subgroups and interactions | b.NA |
| | | (c) Explain how missing data were addressed | c.5 |

| | | | |
|---|---|---|---|
| | | (d) If applicable, explain how loss to follow-up was addressed | d.NA |
| | | (e) Describe any sensitivity analyses | e.8, 10 |
| | | Results | |
| Participants | 13* | (a) Report numbers of individuals at each stage of study—eg numbers potentially eligible, examined for eligibility, confirmed eligible, included in the study, completing follow-up, and analysed | 5 |
| | | (b) Give reasons for non-participation at each stage | 5 |
| | | (c) Consider use of a flow diagram | |
| Descriptive data | 14* | (a) Give characteristics of study participants (eg demographic, clinical, social) and information on exposures and potential confounders | 20 |
| | | (b) Indicate number of participants with missing data for each variable of interest | 5 |
| | | (c) Summarise follow-up time (eg, average and total amount) | 10 |
| Outcome data | 15* | Report numbers of outcome events or summary measures over time | 22 |
| Main results | 16 | (a) Give unadjusted estimates and, if applicable, confounder-adjusted estimates and their precision (eg, 95% confidence interval). Make clear which confounders were adjusted for and why they were included | 22 |
| | | (b) Report category boundaries when continuous variables were categorized | 22, 22 |
| | | (c) If relevant, consider translating estimates of relative risk into absolute risk for a meaningful time period | 10, 11 |
| Other analyses | 17 | Report other analyses done—eg analyses of subgroups and interactions, and sensitivity analyses | 10 |
| Discussion | | | |
| Key results | 18 | Summarise key results with reference to study objectives | 12 |
| Limitations | 19 | Discuss limitations of the study, taking into account sources of potential bias or imprecision. Discuss both direction and magnitude of any potential bias | 14 |
| Interpretation | 20 | Give a cautious overall interpretation of results considering objectives, limitations, multiplicity of analyses, results from similar studies, and other relevant evidence | 12-14 |
| Generalisability | 21 | Discuss the generalisability (external validity) of the study results | 13-14 |
| Other information | | | |
| Funding | 22 | Give the source of funding and the role of the funders for the present study and, if applicable, for the original study on which the present article is based | 15 |

*Give information separately for exposed and unexposed groups.

**Supplementary Table S2.** Univariable associations of baseline characteristics with plasma concentrations of TMAO.

| Variable | Std β (95% CI) | *p* value |
|---|---|---|
| Men, *n* (%) | −0.06 (−0.22; 0.11) | 0.50 |
| Age, years | 0.11 (0.03; 0.19) | **0.007** |
| BMI, kg/m$^2$ | −0.04 (−0.12; 0.05) | 0.39 |
| SBP, mmHg | 0.15 (0.07; 0.23) | **<0.001** |
| DBP, mmHg | −0.04 (−0.12; 0.04) | 0.33 |
| Never smoked, *n* (%) | −0.10 (−0.30; 0.10) | 0.31 |
| Diabetes duration, years | 0.02 (−0.06; 0.10) | 0.60 |
| Macrovascular comp., *n* (%) | 0.17 (0.00; 0.33) | 0.05 |
| Microvascular comp., *n* (%) | 0.19 (0.03; 0.35) | **0.02** |
| Glucose, mg/dL | −0.01 (−0.09; 0.07) | 0.81 |
| HbA1c, % | −0.02 (−0.10; 0.06) | 0.58 |
| TC, mg/dL | 0.08 (0.00; 0.16) | 0.06 |
| HDL-C, mg/dL | 0.01 (−0.07; 0.09) | 0.83 |
| Triglycerides, mg/dL | −0.03 (−0.11; 0.05) | 0.50 |
| BCAA, μmol/L | 0.10 (0.02; 0.18) | **0.01** |
| Serum creatinine, mmol/L | 0.16 (0.09; 0.24) | **<0.001** |
| Albuminuria, *n* (%) | 0.05 (−0.12; 0.22) | 0.53 |

Standardized regression coefficients are shown. Abbreviations: BMI, body mass index; SBP, systolic blood pressure; DBP, diastolic blood pressure; TC, total cholesterol; HDL-C, high-density lipoprotein cholesterol; BCAA, Branched chain amino acids. *P* values <0.05 are highlighted with bold font.

**Supplementary Table S3.** Association of branched chain amino acids with cardiovascular mortality, assessed with Cox Proportional Hazard ratios.

| | BCAA per 1 Ln SD Increment | | T1 | T2 | | T3 | |
|---|---|---|---|---|---|---|---|
| Participants, *n* | 595 | | 194 | 193 | | 208 | |
| Events, *n* | 113 | | 22 | 32 | | 59 | |
| | HR (95 % CI) | *p* value | | HR (95 % CI) | *p* value * | HR (95 % CI) | *p* value ** |
| Crude Model | 0.84 (0.70;1.01) | 0.06 | (ref) | 0.69 (0.44;1.08) | 0.11 | 0.67 (0.43;1.05) | 0.07 |
| Model 1 | 0.99 (0.82;1.21) | 0.95 | (ref) | 0.91 (0.58;1.44) | 0.70 | 1.06 (0.66;1.69) | 0.80 |
| Model 2 | 0.99 (0.81;1.20) | 0.89 | (ref) | 0.84 (0.54;1.33) | 0.46 | 0.95 (0.60;1.52) | 0.83 |
| Model 3 | 0.93 (0.76;1.15) | 0.51 | (ref) | 0.74 (0.46;1.17) | 0.20 | 0.89 (0.54;1.45) | 0.63 |
| Model 4 | 0.97 (0.78;1.20) | 0.74 | (ref) | 0.73 (0.47;1.24) | 0.27 | 1.04 (0.63;1.73) | 0.86 |
| Model 5 | 0.90 (0.72;1.12) | 0.34 | (ref) | 0.72 (0.44;1.18) | 0.19 | 0.88 (0.52;1.49) | 0.64 |

Data are presented as hazard ratios (HRs) with 95% confidence intervals (CIs) and *p* values. *p* values correspond to the comparison between T1 vs T2 (*) and T1 vs T3 (**). Model 1. Model adjusted for age + sex. Model 2. Model 1 + T2D duration + smoking + macrovascular complications. Model 3. Model 2 + SBP + HbA1c + TC + HDL-C+ TG. Model 4. Model 3 + albuminuria. Model 5. Model 4 + reduced eGFR (<60 mL/min/1.73 m$^2$) + TMAO. Abbreviations: T2D, type 2 diabetes; SBP, systolic blood pressure; TC, total cholesterol; HDL-C, high-density lipoprotein cholesterol; TG, triglycerides; BCAA, Branched chain amino acids; eGFR, estimated glomerular filtration rate. (ref) indicates the group of reference.

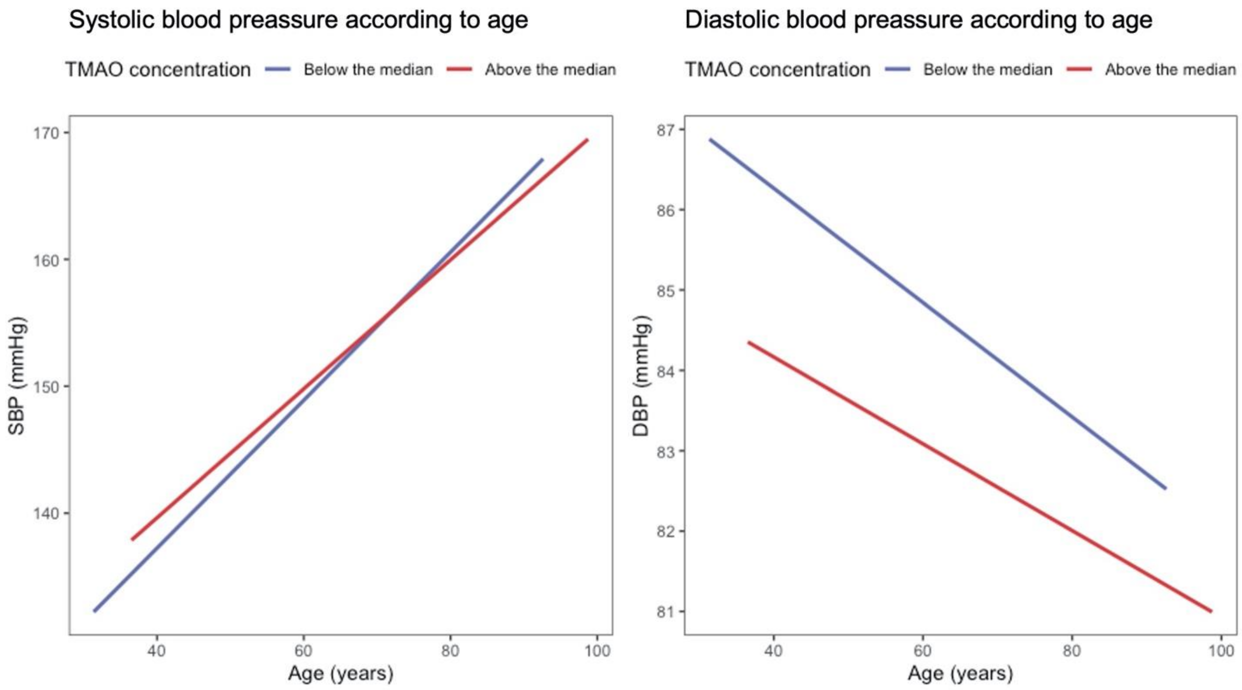


**Supplementary Figure S1.** Association of age with blood pressure by plasma concentrations of Trimethylamine N-oxide. The left panel depicts the association of age with systolic blood pressure in individuals above and below the median. The right panel depicts the association of age with diastolic blood pressure in individuals above and below the median.

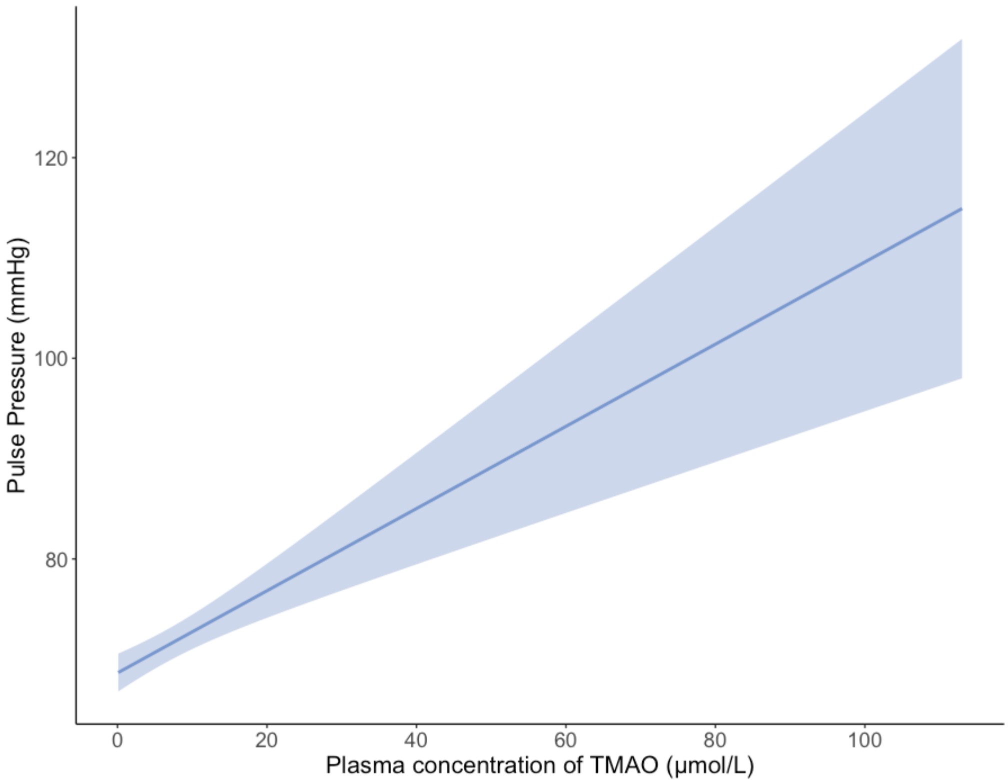


**Supplementary Figure S2.** Association of pulse pressure with plasma concentrations of Trimethylamine N-oxide. Higher concentrations of TMAO in plasma are associated with elevated pulse pressure. Std. β= 0.10 (95% CI 0.06; 0.14), $p$<0.001.
